# Semiartificial Photosynthetic Nanoreactors for H_2_ Generation

**DOI:** 10.1021/jacs.4c12311

**Published:** 2024-12-03

**Authors:** Huijie Zhang, Jan Jaenecke, Imogen L. Bishara-Robertson, Carla Casadevall, Holly J. Redman, Martin Winkler, Gustav Berggren, Nicolas Plumeré, Julea N. Butt, Erwin Reisner, Lars J. C. Jeuken

**Affiliations:** † Leiden Institute of Chemistry, Leiden University, PO box 9502, 2300 RA Leiden, The Netherlands; ‡ Campus Straubing for Biotechnology and Sustainability, Technical University of Munich, Uferstrasse 53, 94315 Straubing, Germany; § Yusuf Hamied Department of Chemistry, University of Cambridge, Lensfield Road, Cambridge CB2 1EW, United Kingdom; ¶ Department of Chemistry-Ångström laboratory, Molecular Biomimetics, Uppsala University, Box 523, 75120 Uppsala, Sweden; ⊥ School of Chemistry and School of Biological Sciences, University of East Anglia, Norwich Research Park, Norwich NR47TJ, United Kingdom

## Abstract

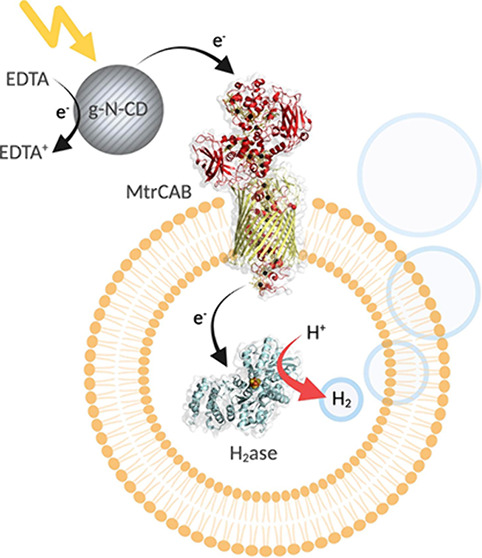

A relatively unexplored energy source in synthetic cells is transmembrane electron transport, which like proton and ion transport can be light driven. Here, synthetic cells, called nanoreactors, are engineered for compartmentalized, semiartificial photosynthetic H_2_ production by a *Clostridium beijerinckii* [FeFe]-hydrogenase (H_2_ase). Transmembrane electron transfer into the nanoreactor was enabled by MtrCAB, a multiheme transmembrane protein from *Shewanella oneidensis* MR-1. On illumination, graphitic nitrogen-doped carbon dots (g-N-CDs) outside the nanoreactor generated and delivered photoenergized electrons to MtrCAB, which transferred these electrons to encapsulated H_2_ase without requiring redox mediators. Compartmentalized, light-driven H_2_ production was observed with a turnover frequency (TOF_H2ase_) of 467 ± 64 h^–1^ determined in the first 2 h. Addition of the redox mediator methyl viologen (MV) increased TOF_H2ase_ to 880 ± 154 h^–1^. We hypothesize that the energetically “uphill” electron transfer step from MtrCAB to H_2_ase ultimately limits the catalytic rate. These nanoreactors provide a scaffold to compartmentalize redox half reactions in semiartificial photosynthesis and inform on the engineering of nanoparticle–microbe hybrid systems for solar-to-chemical conversion.

Synthetic cells, also known as artificial cells or protocells, are engineered systems, often lipid vesicles, that aim to mimic important and complex functions in biology.^1^ Controlling transport of reactants across the lipid membrane provides synthetic cells with the key ability to harvest and utilize energy.^2,3^ For instance, transmembrane electrochemical gradients can be formed by transporting or pumping protons, and used to drive energetic uphill reactions such as ATP synthesis.^4^ Similarly, reactants can be transported into the synthetic cell, where they are converted by biocatalysts to produce ATP.^2^ Synthetic cells can also acquire energy from light using photosynthetic principles.^5−8^ In artificial photosynthesis, lipid vesicles have been used to solve solubility issues of inorganic catalysts for solar energy conversion in water, for example H_2_O oxidation,^9−11^ H_2_ generation^12−14^ and CO_2_ reduction.^15−18^ In these systems, photosensitizers and catalysts are typically coembedded into the fluid membrane to enhance electron transfer efficiency. However, to our knowledge, none of these systems rely on a transmembrane electron conduit to transport photoelectrons into the synthetic cell for solar fuel synthesis.

Natural photosynthesis in plant cells occurs across the thylakoid membrane, compartmentalizing two redox half-reactions while minimizing chemical back reactions.^19^ When mimicking this property in a synthetic cell, one needs to engineer a system with two half-reactions in different nano- or microcompartments, which require electron exchange across the membrane. Here, we developed a synthetic cell, henceforth referred to as a “nanoreactor”, using a multiheme protein complex MtrCAB from *Shewanella oneidensis* MR-1^20−22^ for transmembrane electron transfer. Combined with graphitic nitrogen-doped carbon dots (g-N-CDs)^23,24^ as a photosensitizer, a photoactive, compartmentalized nanoreactor platform was created (Figure 1). We previously showed that g-N-CD photoreduces MtrC,^23^ enabling transmembrane photoelectron transfer through MtrCAB.^25,26^

**Figure 1:**
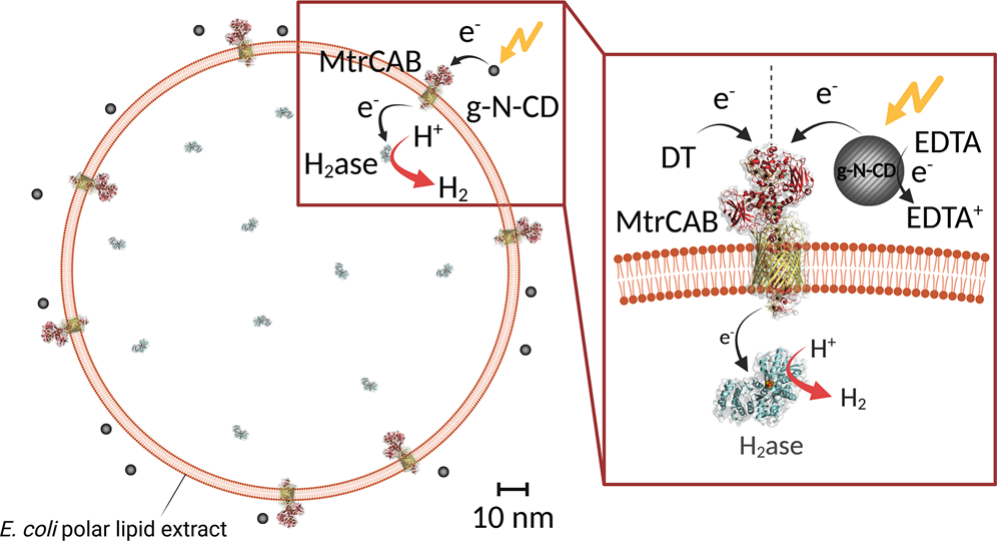
Illustration of the nanoreactors used for semiartificial photobiological hydrogen generation. H_2_ase is encapsulated within a lipid-based nanoreactor containing the transmembrane electron transfer protein MtrCAB. H_2_ generation is driven by chemical reductant dithionite (DT) or photocatalytically by irradiation of extravesicular g-N-CD which leads to the donation of photoexcited electrons into the nanoreactor.

MtrCAB has previously been used in nanoreactors that photoreduce N_2_O to N_2_ via encapsulated N_2_O reductase,^27^ but the formation of a catalytic, fuel-forming nanoreactor has not yet been demonstrated. To transfer electrons from MtrCAB to N_2_O reductase, the electron mediator, methyl viologen (MV), was required, which is lipid-membrane permeable in its reduced form.^27−29^ To create a nanoreactor for photosynthetic fuel generation, we encapsulated *Cb*A5H, a [FeFe]-hydrogenase from *Clostridium beijerinckii*.^30^ By quantifying the components and catalytic rate of the nanoreactors, the rate limiting step of the system was characterized for future optimization.

MtrCAB nanoreactors encapsulating H_2_ase (||MtrCAB/H_2_ase||) were prepared as described in the Experimental section. A nanoreactor control with only MtrCAB (||MtrCAB||) mixed with nanoreactors containing only H_2_ase (||MtrCAB|| + ||H_2_ase||) confirmed that no H_2_ase is located outside the nanoreactors (see below). The nanoreactors exhibit a hydrodynamic diameter of 130 ± 13 nm, as determined by dynamic light scattering (Figure S1). The number of reconstituted MtrCAB in the nanoreactors was determined via UV–vis absorption spectroscopy using the Soret peak at 410 nm (Figure 2a). MtrCAB concentration was determined to be 13 nM for a 1.8 nM nanoreactor solution: ∼7 MtrCAB per nanoreactor (Supporting Information). MtrB and MtrA were visualized by denaturing polyacrylamide gel electrophoresis (SDS-PAGE), showing bands with apparent molecular weights of ∼75 and ∼33 kDa (Figure 2b). MtrC and H_2_ase have comparable sizes, ∼70 kDa, and thus a peroxidase-linked heme stain was used to confirm the presence of cytochromes (Figure 2c). The number of H_2_ase per nanoreactor was quantified by strep-tag Western blot (Figures 2d and S2) to be 0.20 μM for an 18 nM nanoreactor solution, corresponding to approximately 11 H_2_ase per nanoreactor. MtrCAB and H_2_ase are observed as two individual bands on native-PAGE (Figure S3), indicating that MtrCAB and H_2_ase do not form a tight complex.

**Figure 2:**
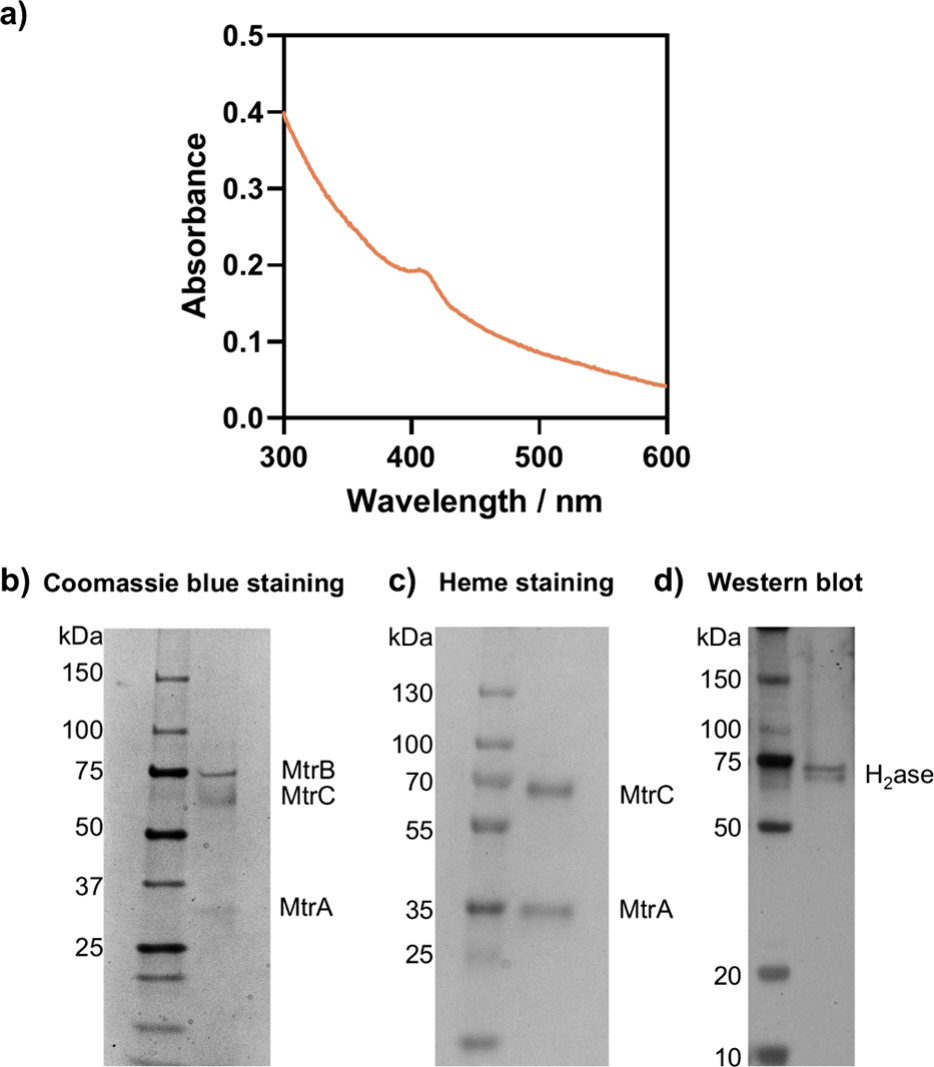
Characterization of nanoreactor. a) UV–vis absorbance of 1.8 nM ||MtrCAB/H_2_ase|| in 20 mM MOPS, 30 mM Na_2_SO_4_, pH 7.4. SDS-PAGE gel image of b) Coomassie stained and c) peroxidase-linked heme stained for ||MtrCAB/H_2_ase||. d) Strep-tag Western blot image for ||MtrCAB/H_2_ase||.

The electron transfer pathway in the nanoreactor system was investigated using sodium dithionite (DT) as an external chemical reducing agent (Figure 1). A Clark electrode (Figure S4) and gas chromatography (GC) were employed to quantify H_2_ generation. For ||H_2_ase|| or a mixed solution of ||MtrCAB||+||H_2_ase||, no H_2_ formation was detected upon the addition of DT (Figure 3). In contrast, a significant amount of H_2_ is generated in the ||MtrCAB/H_2_ase|| nanoreactors, confirming direct electron transfer from DT-reduced MtrCAB to encapsulated H_2_ase. For ||MtrCAB/H_2_ase|| a turnover number for H_2_ase (TON_H2ase_) of approximately 2000 after 2 h was determined. Because of the small lumen volume of the nanoreactors, H^+^ might be quickly consumed. To verify if the system is limited by the local internal pH, a protonophore, carbonyl cyanide *m*-chlorophenyl hydrazone (CCCP), was added to exchange protons across the lipid bilayer. H_2_ generation in ||MtrCAB/H_2_ase|| with and without CCCP was comparable (Figure 3b), indicating that the activity is not limited by slow H^+^ transfer. We hypothesize that MtrCAB might facilitate H^+^ transfer during the reaction, as proton transport has been suggested to be coupled with electron transfer in the outer-membrane MtrCAB of *S. oneidensis* MR-1.^20,31^

**Figure 3:**
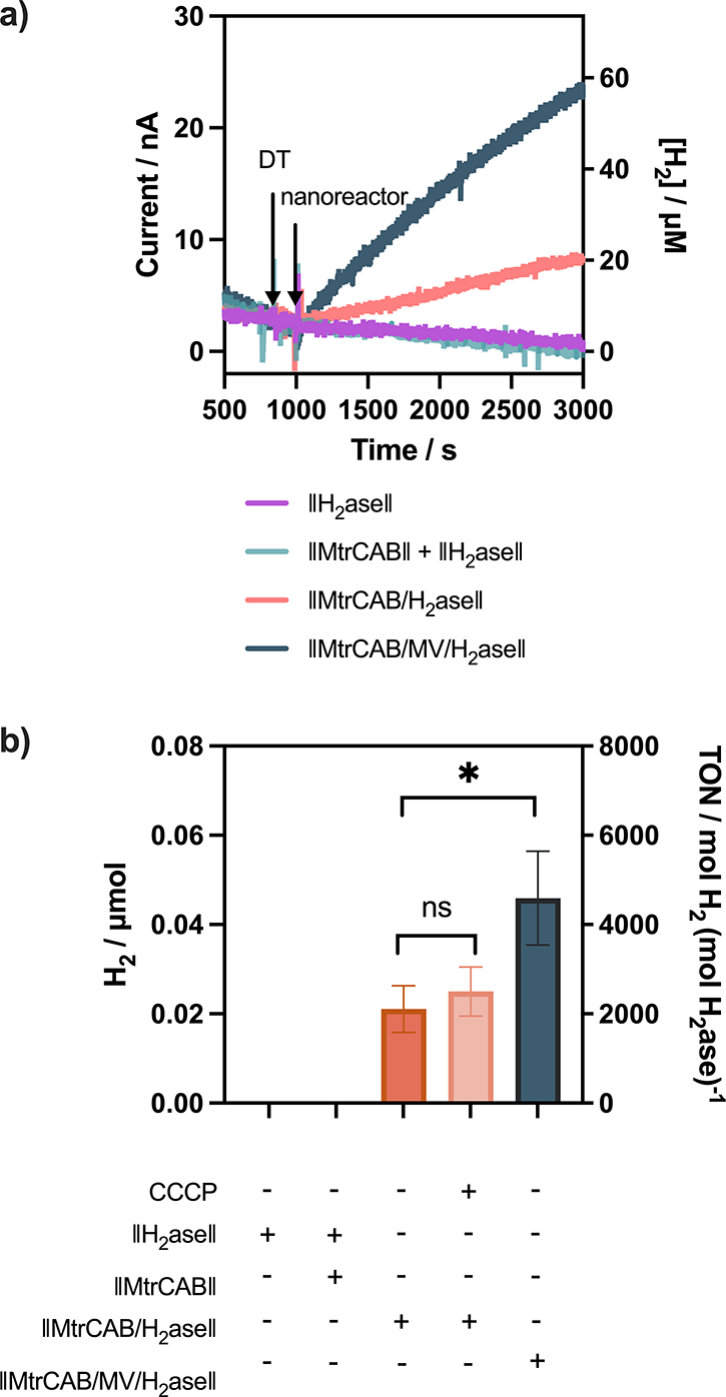
DT-driven H_2_ generation. a) H_2_ generation in solution detected by Clark electrode. DT and different nanoreactors were added as indicated in the figure. b) H_2_ generation detected in the reaction headspace after 2 h by GC upon addition of DT for different nanoreactor preparations, as indicated. All experiments were performed with 500 μL reaction volume (with a 4 mL headspace for GC), 2 nM nanoreactor, 10 mM DT, 20 μM CCCP, 20 mM MOPS, 30 mM Na_2_SO_4_, pH 7.4. The Clark electrode data shows representative samples and the GC data are an average of 3 data sets, with the standard deviation given by error bars and * signifies *p* < 0.05.

The turnover frequency of “free” H_2_ase was determined to be ∼55 s^–1^ by GC using 100 mM DT and 10 mM MV under the same conditions as used for the nanoreactors. This is many orders of magnitude higher than the rates observed for ||MtrCAB/H_2_ase|| (Table 1). Given that the transmembrane electron transfer rate for MtrCAB is on the order of 10^3^ s^–1^,^22^ and reduction of MtrCAB by DT is also very fast, it follows that the electron transfer from MtrCAB to H_2_ase is the most likely rate limiting step. To check whether the interaction between MtrCAB and H_2_ase is limiting performance, we increased the amount of H_2_ase in the nanoreactor and, in a separate experiment, coencapsulated MV^2+^ in the nanoreactors. Increasing the concentration of H_2_ase in the nanoreactor has no effect on H_2_ evolution, confirming that H_2_ase activity is not rate limiting (Figure S5). Reduction of MV^2+^ was verified by UV–vis spectroscopy after the addition of DT (Figure S6). For ||MV/H_2_ase||, no MV^+•^ was observed with UV–vis spectroscopy after addition of (membrane-impermeable) DT. However, reduced MV^+•^ was observed after the nanoreactors were lysed with Triton X-100, confirming that MV was encapsulated in the nanoreactors (Figure S6a). Encapsulating MV (||MtrCAB/MV/H_2_ase||) roughly doubles the rate of H_2_ formation, but the H_2_ formation rate remains far below that of TOF_H2ase_ for free H_2_ase (Figure 3, Table 1).

**Table 1:** Summary of the Photocatalytic Performance of the Nanoreactors

	TOF_H2ase_/h^–1^	
	DT	Light-driven
||MtrCAB/H_2_ase||	1054 ± 261	467 ± 64
||MtrCAB/MV/H_2_ase||	2295 ± 525	880 ± 154

TOF_H2ase_ (turnover frequency normalized against H_2_ase) is calculated based on the H_2_ generation in the first 2 h for a 2 nM nanoreactor sample (20 mM MOPS, 30 mM Na_2_SO_4_, pH 7.4 was used for DT (10 mM) driven H_2_ generation, 50 mM sodium phosphate buffer, pH 7.4, 100 mM EDTA, 150 μg/mL g-N-CD was used for light-driven H_2_ generation).

We propose that the lower TOF_H2ase_ in the nanoreactor compared to that in free H_2_ase is due to the electron transfer steps from MtrCAB to H_2_ase. The 20 hemes in MtrCAB protein are reported to have a distribution in redox potentials (*E*^0′^), between 0 and −0.4 V *vs* standard hydrogen electrode (SHE),^21^ while the potential with which electrons either enter or exit MtrCAB in *S. oneindensis* MR-1 *in vivo* has been measured to be about −0.2 V *vs* SHE.^32−34^ Similar to [FeFe]-hydrogenase from *Clostridium pasteurianum* (CpI),^35^ we expect electrons enter *Cb*A5H H_2_ase via the distal [4Fe-4S] cluster and then transfer via the additional accessory [FeS] clusters to the H-cluster. Although the reduction potential of the [4Fe-4S] cluster is unknown, the reduction potential of the 2H^+^/H_2_ equilibrium at pH 7.4 (−0.44 V *vs* SHE) or MV (−0.45 V *vs* SHE)^36^ are more negative than MtrCAB. Indeed, when reducing MV encapsulated in nanoreactors containing MtrCAB (||MtrCAB/MV||), only a fraction of the MV is reduced, confirming an equilibrium is formed between reduced MtrCAB and MV^2+^/MV^+•^ (Figure S6b). In the ||MtrCAB/MV/H_2_ase|| nanoreactors, almost no reduced MV^+•^ is observed in the presence of excess DT (Figure S6c), indicating that MV^+•^ oxidation by H_2_ase is faster than MV^2+^ reduction by MtrCAB.

To determine if the electron transfer between MtrCAB and H_2_ase is rate limiting because *E*^0'^_MtrCAB_ > *E*^0'^_H2ase_, we measured the H_2_ generation of ||MtrCAB/MV/H_2_ase|| at pH 7, pH 7.4, and pH 8 (Figure S7). The redox potential of MtrC is pH-dependent, increasing 47 mV per unit increase in pH (Figure S8), and we expect MtrCAB to exhibit a similar behavior. Hence, the difference in reduction potential between MtrCAB and 2H^+^/H_2_ remains approximately constant with pH. The results showed that the H_2_ evolution rate is the same or just slightly increases with rising pH, reflecting the pH-dependent activity profile of *Cb*A5H.^30^ This observation supports our hypothesis that electron transfer from MtrCAB to H_2_ase is rate limiting.

With the ||MtrCAB/H_2_ase|| nanoreactors established, g-N-CDs were used as a photosensitizer for light-driven hydrogen formation (Figure 4). A TON_H2ase_ of 938 ± 127 was observed after 2 h of irradiation, within the same order of magnitude as using chemical reductant DT. As expected, no H_2_ was detected with either ||H_2_ase|| or ||MtrCAB||+||H_2_ase|| controls or when EDTA, g-N-CD, light, or ||MtrCAB/H_2_ase|| was absent. This demonstrates that the photoenergized electrons in g-N-CD are transferred via MtrCAB to H_2_ase, which catalyzes H_2_ generation. Hydrogen generated by g-N-CD/||MtrCAB/H_2_ase|| seems to increase for at least 5 h, although further increases after 1 h are not statistically significant (Figure 5). Finally, similar to the DT reduced system, coencapsulation of MV in the light-driven nanoreactor only doubles the H_2_ evolution rate (Figure 4, Table 1). We thus conclude that even in the light-driven system, electron transfer from MtrCAB to H_2_ase remains at least partly limiting. The lower TOF_H2ase_ for the light-driven reactions compared to the DT reduction indicates that photoreduction of MtrCAB by g-N-CD is also partly rate limiting, although this effect is small relative to the uphill electron transfer from MtrCAB to H_2_ase.

**Figure 4:**
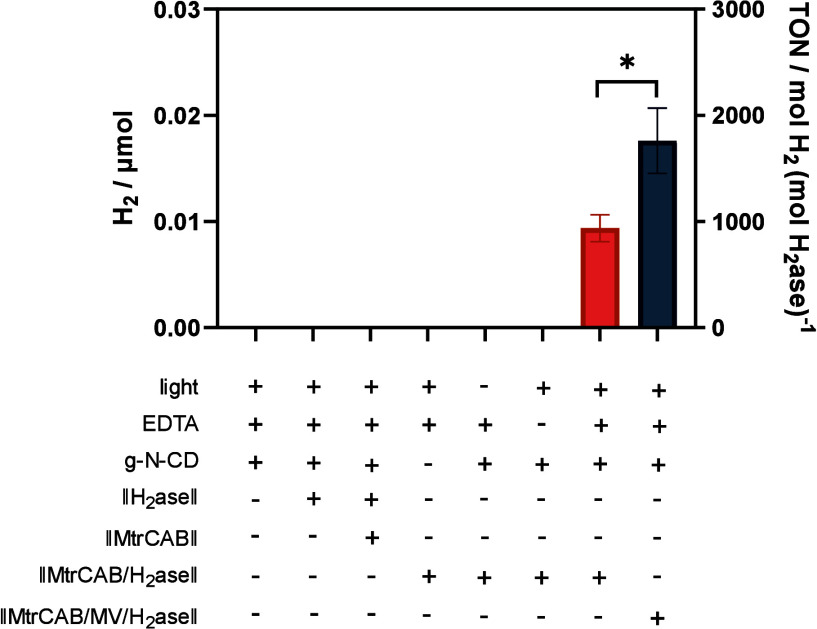
Photocatalytic H_2_ generation. H_2_ generation detected by GC after 2 h of illumination. 500 μL reaction volume in 4.5 mL glass vial (4 mL in the headspace), ∼2 nM nanoreactor, 100 mM EDTA, 150 μg/mL g-N-CD, 50 mM sodium phosphate buffer, pH 7.4. The samples were illuminated by 6200K white LED with an intensity of 29 mW/cm^2^ at 20 °C. Error bars show standard deviation (*n* = 3).

**Figure 5:**
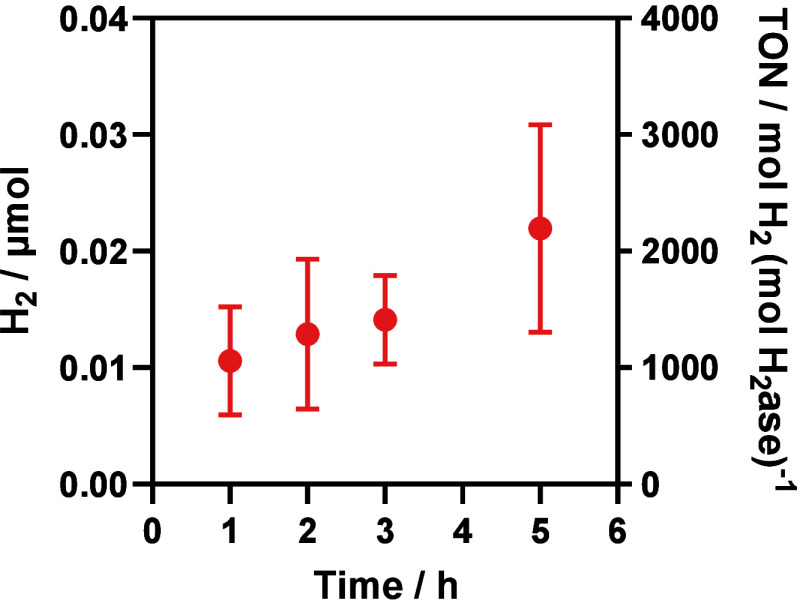
Time-dependent photocatalytic H_2_ generation of ||MtrCAB/H_2_ase|| detected by gas chromatography. 500 μL reaction volume in 4.5 mL glass vial, 2 nM nanoreactor, 100 mM EDTA, 150 μg/mL g-N-CD, 50 mM sodium phosphate buffer, pH 7.4. The samples were illuminated by 6200K white LED with an intensity of 29 mW/cm^2^ at 20 °C. Error bars show standard deviation (*n* = 3).

In conclusion, a semiartificial photosynthetic nanoreactor has been constructed for H_2_ production. Light-induced electron transfer from photosensitizer g-N-CD, *via* MtrCAB, to the H_2_ase inside the nanoreactor fuels H_2_ generation without the need for redox mediators. This shows that MtrCAB and H_2_ase directly exchange electrons. A key rate limiting step was identified as electron transfer from MtrCAB to H_2_ase. We propose that the more positive redox potential of MtrCAB renders electron transfer from MtrCAB (directly or *via* MV) to H_2_ase rate limiting. Our results underline the importance of redox potentials in nanoreactor systems when synthesizing fuels with a low redox potential such as hydrogen.

## Supporting Information Available

Additional experimental details, materials, and methods, calculations used to determine the number of MtrCAB and H_2_ase per nanoreactor, dynamic light scattering of nanoreactors, Western Blot and native PAGE data to determine H_2_ase content and MtrCAB-H_2_ase interaction, calibration of the Clark electrode, control data of nanoreactors with H_2_ase or MV, data of ||MtrCAB/MV/H_2_ase|| and MtrC at different pH (PDF)
