# Supplementary material for: Semiartificial Photosynthetic Nanoreactors for H2 Generation
Source: J Am Chem Soc. 2024 Dec 3;146(50):34260–4. doi: 10.1021/jacs.4c12311 (PMC11664579; doi:10.1021/jacs.4c12311)
Supplement: Supplementary file 1 — ja4c12311_si_001.pdf [file ja4c12311_si_001.pdf]

Supplementary Information for

Semi-artificial Photosynthetic Nanoreactors for H_2 Generation

Huijie Zhang,[1] Jan Jaenecke,[2] Imogen L. Bishara-Robertson,[1] Carla. Casadevall, †[3] Holly J. Redman,[4] Martin Winkler,[2] Gustav Berggren,[4] Nicolas Plumeré,[2] Julea N. Butt,[5] Erwin Reisner[3] and Lars J. C. Jeuken*[1]

1. Leiden Institute of Chemistry, Leiden University, PO box 9502, 2300 RA Leiden, The Netherlands. Email: l.j.c.jeuken@lic.leidenuniv.nl.
2. Campus Straubing for Biotechnology and Sustainability, Technical University Munich, Uferstraße 53, 94315, Straubing, Germany
3. Yusuf Hamied Department of Chemistry, University of Cambridge, Lensfield Road, Cambridge CB2 1EW, United Kingdom
4. Department of Chemistry—Ångström laboratory, Molecular Biomimetics, Uppsala University, Box 523, 75120 Uppsala, Sweden.
5. School of Chemistry and School of Biological Sciences, University of East Anglia, Norwich Research Park, Norwich, NR47TJ, United Kingdom

† Present address: Institute of Chemical Research of Catalonia (ICIQ), The Barcelona Institute of Science and Technology, Avinguda dels Països Catalans, 16, 43007 Tarragona, Spain.; Department of Physical and Inorganic Chemistry, University Rovira i Virgili (URV), C/ Marcel.lí Domingo, 1, 43007 Tarragona, Spain.

Experimental section

Materials

MtrCAB was purified from *Shewanella oneidensis* MR-1 as described previously.[1] MtrC, which lacks the lipid attachment site found in the wild-type MtrC and includes a C-terminal Strep II affinity tag, was obtained using a previously described method.[1] [FeFe]-Hydrogenase *Cb*A5H was prepared and activated according to the procedure described by Esselborn and coauthors[2] with adaptions which enabled an increase in the production yield of active holoenzyme, as follows. *Cb*A5H expression was induced at an OD of 0.8 instead of 0.4.[3] For *in vitro* maturation apo-hydrogenase was mixed with $2FeH^{MIM}$ at a ratio of 1:3 and incubated for 30 min at RT. Excess $2FeH^{MIM}$ was removed from the holo-*Cb*A5H sample, using 30kDa MWCO spin filters instead of applying a gel filtration step, which minimizes the protein loss. g-N-CD was synthesized as reported.[4] All chemicals were obtained from Merck unless otherwise specified.

Nanoreactor Preparation

5 mg of *E. coli* polar lipid extract (Avanti Polar Lipids) was dispersed in 125 µL of MOPS buffer (20 mM, 30 mM Na_2SO_4, pH 7.4) or 20 mM methyl-viologen (MV) in MOPS buffer by vortex for 15 min at room temperature. 50 µL of 0.5 M octyl glucoside (OG) was added left on ice for 10 min to solubilize the lipid. For the liposomes containing MtrCAB, 30 µL 20 µM MtrCAB was mixed with the lipid/OG solution and left on ice for 10 mins. For the liposomes containing H_2ase, 3.1 µL 400 µM H_2ase was added and left on ice for another 10 mins. To remove OG, 50 mg Bio-Beads SM-2 was added to the samples and incubated on ice for 30 mins. The solution was transferred to fresh Bio-Beads (50 mg) and this process was repeated for 3 times. To recover the formed nanoreactors and remove non-encapsulated H_2ase and MV, if present, the samples were diluted with MOPS buffer to about 60 mL and pelleted by ultracentrifugation in a Ti45 rotor (Beckman) at 42 000 rpm at 4 °C. We note that MV in its oxidized form (MV^{2+}), the redox form used in this preparation, is membrane impermeable and hence can be encapsulated without MV leaking out of the nanoreactor. The supernatant was discarded, the nanoreactors were resuspended in 60 mL MOPS buffer and the ultracentrifugation/resuspension steps were repeated 3 times. The nanoreactors were then resuspended in 250 µL MOPS buffer and centrifuged at 5000 g for about 5 mins to remove any aggregates. The nanoreactor concentration in the resulting samples wase estimated to be 18 nM from their size as determined with dynamic light scattering (DynaPro NanoStar, WYATT Technology) as described below. The nanoreactors used for the light-driven reactions were prepared with sodium phosphate buffer (50 mM, pH 7.4).

The standard H_2ase assay uses sodium dithionite (DT) and the redox mediator MV. However, this standard assay could not be used to distinguish between H_2ase inside the nanoreactor and H_2ase outside the nanoreactor because reduced redox mediator $MV^{+\bullet}$ is able to diffuse through the membrane.[5, 6] Instead, for our negative controls, nanoreactors with only MtrCAB (‖MtrCAB‖) were prepared and mixed with nanoreactors containing only H_2ase (‖MtrCAB‖ + ‖H_2ase‖). These controls confirmed no H_2ase is located outside the nanoreactors as shown in the main text.

To check if the activity of H_2ase was affected by the nanoreactor preparation procedure, a ‖H_2ase‖ sample was lysed with Triton X100, and the activity of the released H_2ase was determined and compared with that of a H_2ase sample that was not subjected to the nanoreactor preparation process. No difference in activity was observed (**Figure S9**).

Electrochemistry measurement with Clark electrode for H_2 detection

A Clark electrode (Hansatech Instruments Ltd) was used for electrochemically detection of H_2 generation. The Clark electrode uses a silver ring as counter/reference electrode and a platinum disc electrode as working electrode. Both electrodes are immersed in 3 M KCl electrolyte solution. A gas-permeable membrane, which encloses the electrode, enables the H_2 generated in the reaction chamber diffuse to the Pt working electrode. The Clark electrode was connected to a potentiostat (AutoLab). Chronoamperometry was used to monitor the current with applied potential at +0.6 V (vs Ag/AgCl, 3 M KCl).

A gas mixture of 5 % H_2 in 95 % Ar was used to calibrate the Clark electrode. The electrode was first polarized and then 5 % H_2 calibration as was flushed through the buffer in the chamber using mass flow controller (Sierra Instruments, accuracy ±1%; typical flow rate 10 ml per minute) till the current signal start to saturate. Then the H_2 was removed by flushing the buffer with 100 % Ar. A calibration curve is shown in Figure S4. The saturated current was used to represent the H_2 concentration of 5 % H_2 equilibrated solution which is 38 μM calculated as following.

The H_2 concentration of the sample equilibrated with 5% H_2 was calculated using the Henry's Law constant $H^{cp} = 7.9 \times 10^{-6} \left(\frac{mol}{m^3 \cdot Pa}\right) = 7.9 \times 10^{-4} \left(\frac{mol}{L \cdot atm}\right)$. H^{cp} was used for Henry's volatility K_H^{pc} defined as $K_H^{pc} = \frac{p}{C} = \frac{1}{H^{cp}}$, where p is the partial pressure of H_2 in the gas phase and C is the concentration of H_2 in the aqueous phase. Thus the concentration of solution equilibrated with 5 % H_2 is $C = \frac{p}{K_H^{pc}} = \frac{0.05\ atm}{1300 \frac{atm \cdot L}{mol}} = 3.8 \times 10^{-5}\ M = 38\ \mu M$

In the chamber, DT and liposomes were added in steps as indicated in the main text. The final reaction mixture contained 10 mM DT, ~2 nM nanoreactor in 500 µL 20 mM MOPS, 30 mM Na_2SO_4, pH 7.4. All the experiments were performed in a MBraun glovebox (O_2 < 1 ppm).

Gas chromatography for H_2 detection

Gas chromatography was conducted using a Shimadzu GC 2010 Plus gas chromatograph, equipped with a Supelco Carboxen 1010 PLOT column (30 m × 0.53 mm). The system was calibrated with H_2 concentrations of 100 ppm, 500 ppm, and 1000 ppm in Ar (Linde Gas). All experiments were carried out in 4.5 mL glass vials. The reaction mixture, with a final volume of 500 µL, was prepared and transferred to the vials inside a MBraun glovebox (O_2 < 1 ppm). For the sodium dithionite driven reaction, ~2 nM nanoreactor, 10 mM DT in 20 mM MOPS, 30 mM Na_2SO_4, pH 7.4 were used. For the light-driven reaction, ~2 nM nanoreactor, 100 mM EDTA, 150 µg/mL g-N-CD, 50 mM sodium phosphate buffer, pH 7.4 were used. The vials were then sealed with caps and removed from the glovebox and illuminated for the given times. Illumination was carried out in PhotoRedOx (HepatoChem) using a 6200K white LED with an intensity of 29 mW/cm^2. At specific time intervals, 1 mL of gas from the vial's headspace was sampled and injected into the gas chromatograph.

To determine the TOF of "free" H_2ase, 100 mM sodium dithionite and 10 mM methyl viologen in 20 mM MOPS, 30 mM Na_2SO_4, pH 7.4 were used. The reaction was carried out in 4.5 mL glass vials at 20 °C without shaking inside the glovebox. After 1 hour, the H_2 produced in the headspace of the vials was quantified using gas chromatography.

Protein film voltammetry of MtrC

Template stripped gold (TSG) working electrodes were prepared according to previously described methods.[7] Briefly, 150 nm gold (99.99%; Goodfellow) was evaporated on silicon wafers (IDB Technology Ltd, UK) and 1.2 cm^2 glass slides were glued to the gold layer with EpoTek 377 for 2 h at 120 °C. The glass slides were detached to expose fresh TSG surfaces that were covered with a self-assembled monolayer (SAM) by incubation overnight at room temperature with a mixture of 0.8 mM 8-mercaptooctanoic acid (in water) and 0.2 mM 1-octanethiol (in ethanol). After incubation, excess thiol was gently washed away with water and the electrode was dried under a nitrogen flow. Protein film electrochemistry was performed in a home-built electrochemical cell with a standard three electrode setup. As the working electrode, the SAM-modified TSG was embedded in a polytetrafluoroethylene (PTFE) holder with a rubber O-ring seal, placed in a glass electrochemical cell container with a platinum wire counter electrode and a saturated silver/silver chloride reference electrode (Ag/AgCl; Radiometer analytical, France) and 2 mL of buffer was added. The working electrode surface

area exposed to the buffer-electrolyte was 0.25 cm^2 . Potentials are converted versus SHE by +0.2 V. To form the MtrC protein film, the electrolyte was removed and the electrode exposed to 50 µL of 1 µM protein solution for 1 min at 20 °C. After rinsing the electrochemical cell more than three times with 2 mL buffer-electrolyte, making sure the electrode remains under fluid throughout, cyclic voltammograms (CVs) were obtained using an Autolab electrochemical analyzer (Ecochemie, Utrecht, Netherlands). 50 mM pH 7 MOPS buffer, pH 8 Tris-HCl buffer, pH 9 Tris-HCl buffer, pH 10 CAPS buffer and pH 11 CAPS buffer were used for the CVs taken at different pH. All experiments were conducted under Ar.

Calculating nanoreactor concentration and number of MtrCAB per nanoreactor

The calculation were performed as described previously.[8] The average molecular weight for *E.coli* polar lipid extract was calculated to be 766.7 Da according to its composition. Dynamic Light Scattering of the liposomes revealed size distributions with a mean diameter of approx. 130 nm. Given that the width of a phospholipid bilayer is approx. 4 nm, meaning the inner leaflets of these liposomes have diameters of approx. 122 nm. Using this diameter and the formula for the surface area of a sphere $A = 4\pi r^2$ the total surface area of a liposome can be estimated,

$$4\pi(65nm)^2 + 4\pi(61nm)^2 = 99801\ \text{nm}^2$$

The number of lipids in one liposome can then be estimated from the average footprint of a phospholipid is approx. 0.7 nm^2

$$99801\ \text{nm}^2 / 0.7\ \text{nm}^2 = 142573$$

Therefore each liposome has an approximate MW of:

$$142573 \times 766.7\ \text{Da} = 109\ \text{MDa}$$

This MW can be used calculate that the total number of liposomes that can be prepared from the 5 mg Polar Lipid Extract used in this study.

$$5\times10^{-3}\text{g} / 1.09\times10^{8}\text{g/mol} = 4.59\ \text{pmol} = 2.7\times10^{13}\ \text{liposome}$$

Therefore, after final resuspension of the liposomes in 250 uL their concentration is estimated as 18 nM.

The MtrCAB concentration was calculated using extinction coefficient of 2000 mM cm^{-1} at 410 nm in the UV-vis spectrum after subtracting the spectral contribution from liposome scattering.

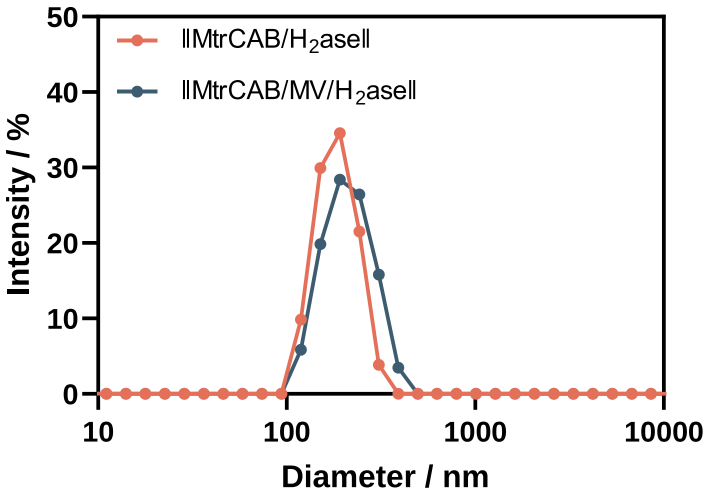

Figure S1. Dynamic light scattering of ‖MtrCAB/H_2ase‖ nanoreactors with and without MV encapsulated.

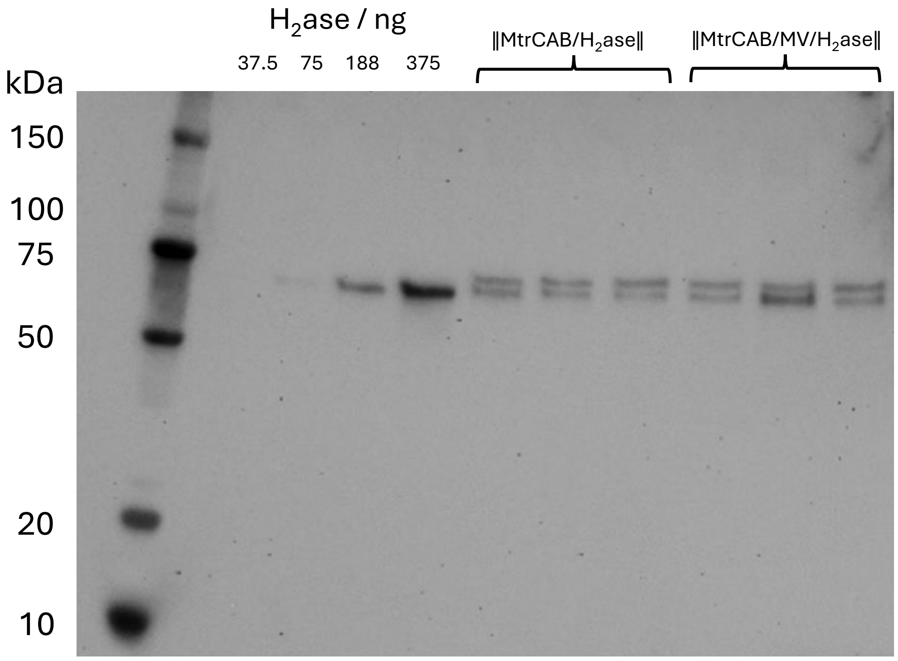

Figure S2. Strep-tag western blot image for H_2ase (37.5, 75, 188 and 375 ng), ‖MtrCAB/H_2ase‖ and ‖MtrCAB/MVH_2ase‖.

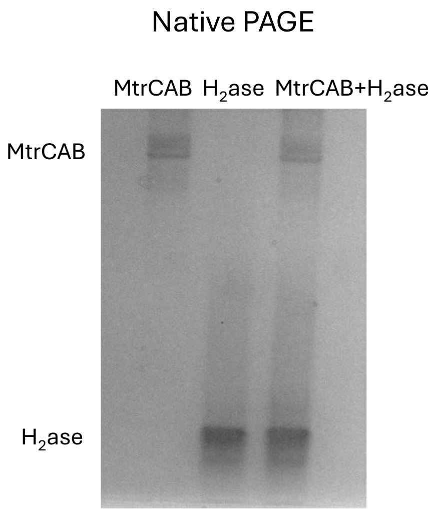

Figure S3. Native-PAGE images visualized by Coomassie Stain of MtrCAB, H_2ase and mixture of MtrCAB and H_2ase (in 1:1 molar ratio).

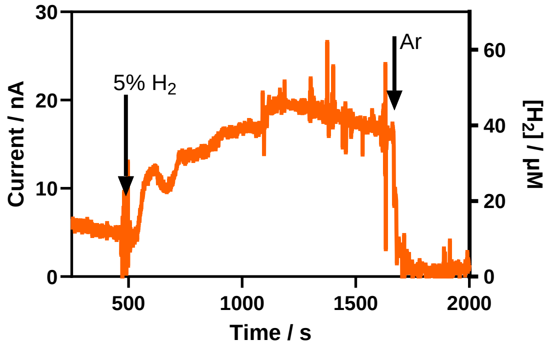

Figure S4. Calibration of the Clark electrode with 5% H_2/ 95 % Ar gas. The solution in the chamber was flushed with 5% H_2/ 95 % Ar gas and 100 % Ar as indicated in the figure. The current is converted to H_2 concentration by using the current value before switching the gas to 100 % Ar to represent the H_2 concentration of the solution equilibrated with 5 % H_2 (38 µM).

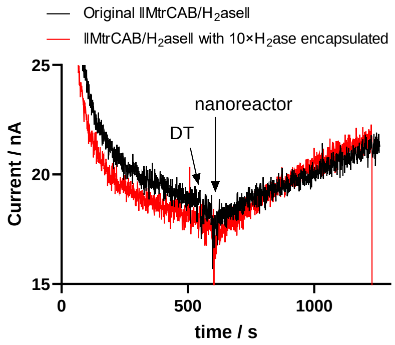

Figure: S5. H_2 generation in solution by ‖MtrCAB/H_2ase‖ nanoreactors**,** detected by Clark electrode. DT and different nanoreactors were added as indicated. The nanoreactor were prepared using the 'standard' protocol in the main manuscript (black lines) and using 10 times the concentration of H_2ase (red line). Experiments were performed with 500 µL reaction volume, 10 mM sodium dithionite (DT), 20 mM MOPS, 30 mM Na_2SO_4, pH 7.4.

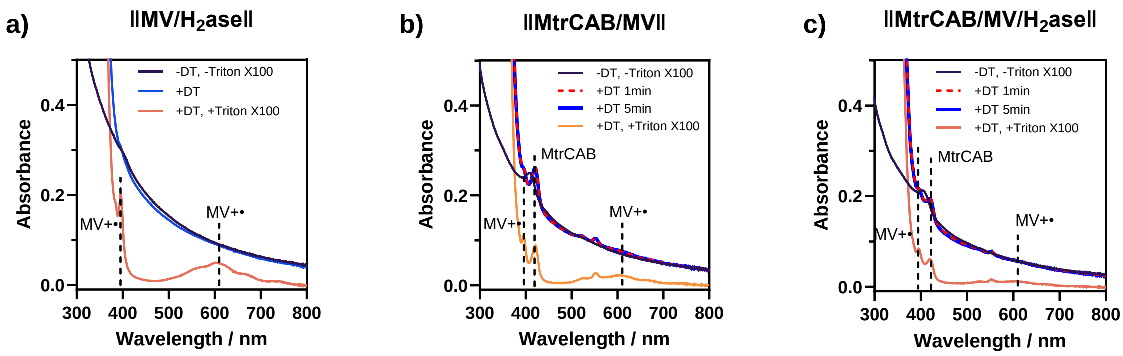

Figure S6. UV–vis absorption spectra of **a)** ‖MV/H_2ase‖, **b)** ‖MtrCAB/MV‖ and **c)** ‖MtrCAB/MV/H_2ase‖ before and after consecutive addition of DT and Triton X100. ~2 nM proteoliposome in 20 mM MOPS, 30 mM Na_2SO_4, pH 7.4. Reduced MV ($MV^{+\cdot}$) has an intense adsorption at 606 nm (ε_{606nm} = 13.7 $mM^{-1}cm^{-1}$).[9]

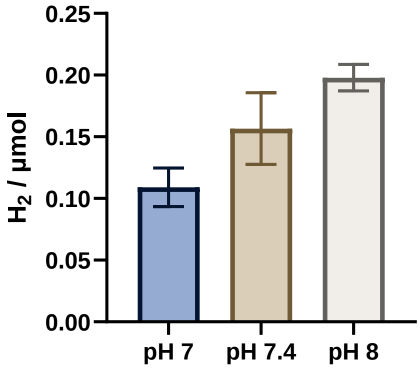

Figure S7. Sodium dithionite (DT)-driven H_2 generation of ‖MtrCAB/MV/H_2ase‖ at pH 7, pH 7.4 and pH 8. All experiments were performed with 500 µL reaction volume with 4 mL in headspace. 2 nM nanoreactor, 10 mM DT, 20 mM MOPS, 30 mM Na_2SO_4.

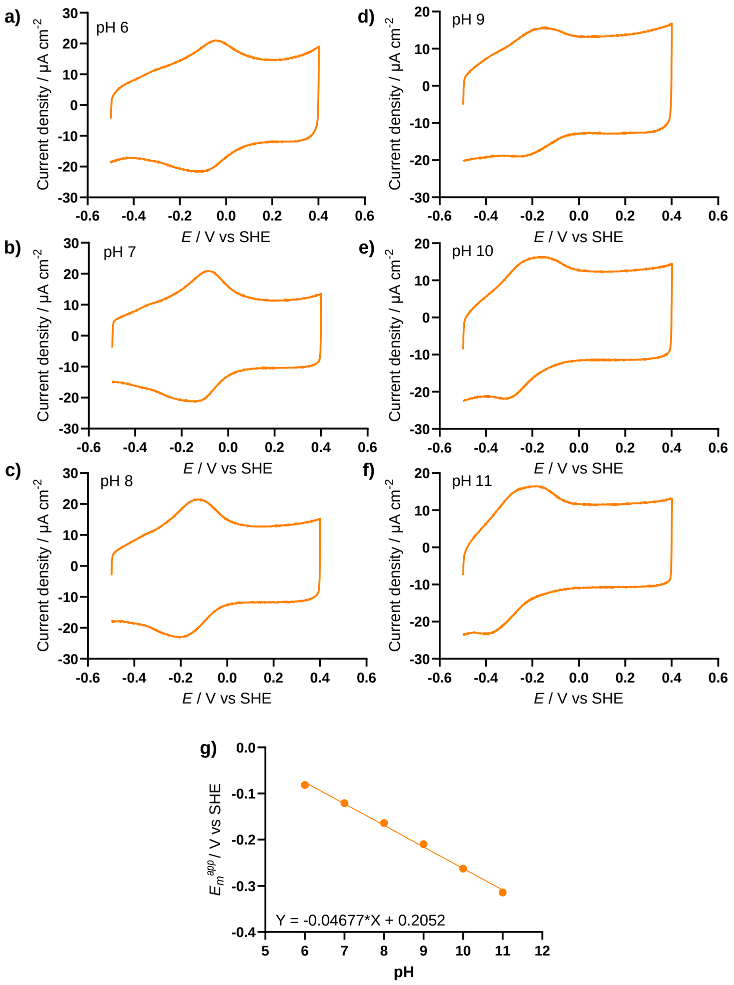

Figure S8. Cyclic voltammograms of MtrC measured at **a)** pH 6, **b)** pH 7, **c)** pH 8, **d)** pH 9, **e)** pH 10 and **f)** pH 11. **g)** the apparent mid-point potential (E_m^{app}, the average of the peak potentials in the CV) at different pH determined from a) to f). E_m^{app} shifts 47 mV per pH unit. Scan rate is 5 V s^{-1}.

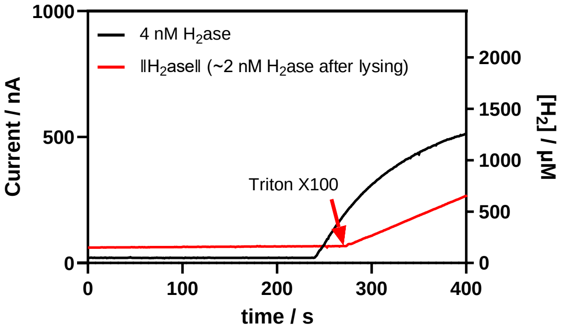

Figure S9: Activity of H_2ase measured with a Clark electrode for 4 nM H_2ase compared to ~2 nM ǁH_2aseǁ after the H_2ase is released from nanoreactor by Triton X100. Experiments were performed with 500 µL reaction volume, 10 mM sodium dithionite (DT), 1 mM methyl viologen, 20 mM MOPS, 30 mM Na_2SO_4, pH 7.4. Activities of H_2ase in this experiment were determined as 1875 s^{-1} for H_2ase and 1827 s^{-1} for the Triton X100 released ǁH_2aseǁ.

References

1. Lockwood, C. W. J.; van Wonderen, J. H.; Edwards, M. J.; Piper, S. E. H.; White, G. F.; Newton-Payne, S.; Richardson, D. J.; Clarke, T. A.; Butt, J. N., Chapter Ten - Membrane-spanning electron transfer proteins from electrogenic bacteria: Production and investigation. In *Methods in Enzymology*, Armstrong, F., Ed. Academic Press: 2018; Vol. 613, pp 257-275.

2. Esselborn, J.; Lambertz, C.; Adamska-Venkatesh, A.; Simmons, T.; Berggren, G.; Noth, J.; Siebel, J.; Hemschemeier, A.; Artero, V.; Reijerse, E. Spontaneous activation of [FeFe]-hydrogenases by an inorganic [2Fe] active site mimic. *Nat. Chem. Biol.* **2013,** *9*, 607-609.

3. Kuchenreuther, J. M.; Grady-Smith, C. S.; Bingham, A. S.; George, S. J.; Cramer, S. P.; Swartz, J. R. High-yield expression of heterologous [FeFe] hydrogenases in Escherichia coli. *PLOS One* **2010,** *5*, e15491.

4. Martindale, B. C.; Hutton, G. A.; Caputo, C. A.; Prantl, S.; Godin, R.; Durrant, J. R.; Reisner, E. Enhancing light absorption and charge transfer efficiency in carbon dots through graphitization and core nitrogen doping. *Angew. Chem. Int. Ed.* **2017,** *129*, 6559-6563.

5. Anderson, R. F.; Patel, K. B. Intracellular and extracellular radiosensitization of *Serratia marcescens* by bipyridinium compounds. *Radiat. Res.* **1979,** *79*, 169-176.

6. Jones, R. W.; Garland, P. B. Sites and specificity of the reaction of bipyridylium compounds with anaerobic respiratory enzymes of Escherichia coli. Effects of permeability barriers imposed by the cytoplasmic membrane. *Biochem. J.* **1977,** *164*, 199-211.

7. Stamou, D.; Gourdon, D.; Liley, M.; Burnham, N. A.; Kulik, A.; Vogel, H.; Duschl, C. Uniformly flat gold surfaces: Imaging the domain structure of organic monolayers using scanning force microscopy. *Langmuir* **1997,** *13*, 2425-2428.

8. Piper, S. E.; Edwards, M. J.; Van Wonderen, J. H.; Casadevall, C.; Martel, A.; Jeuken, L. J.; Reisner, E.; Clarke, T. A.; Butt, J. N. Bespoke biomolecular wires for transmembrane electron transfer: Spontaneous assembly of a functionalized multiheme electron conduit. *Front. microbiol.* **2021**, 2200.

9. Watanabe, T.; Honda, K. Measurement of the extinction coefficient of the methyl viologen cation radical and the efficiency of its formation by semiconductor photocatalysis. *J. Phys. Chem.* **1982,** *86*, 2617-2619.
